# Spectrophotometric Determination of the Antidepressants Sertraline and Paroxetine HCl using 2,4-Dinitrofluorobenzene

**Published:** 2010-09

**Authors:** M. I. Walash, F. Belal, N. El-Enany, H. El-Mansi

**Affiliations:** *Department of Analytical Chemistry, Faculty of Pharmacy, University of Mansoura, Mansoura, 35516, Egypt*

**Keywords:** sertraline, paroxetine HCl, 2,4-dinitrofluorobenzene, spectrophotometry, dosage forms

## Abstract

A simple and sensitive spectrophotometric method was developed for the determination of each of sertraline (SER) and paroxetine HCl (PXT) in dosage forms. The method is based upon reaction of PXT and SER with 2,4-dinitrofluorobenzene (DNFB) to form colored products. The absorbance of the products were measured at 375and 390 nm for SER and PXT respectively. The absorbance concentration plots were rectilinear over the concentration rang of 1-10 and 2-20 μg/mL with lower detection limits (LOD) of 0.11 and 0.28 μg/mL and quantification limits (LOQ) of 0.32 and 0.85 μg/mL for SER and PXT, respectively. The developed method was successfully applied for the determination of SER and PXT in dosage forms. The common excipients and additives did not interfere in their determinations. There was no significant difference between the results obtained by the proposed and the reference methods regarding Student t-test and the variance ratio F-test respectively. A proposal of the reaction pathway was postulated.

## INTRODUCTION

**Figure 1:**
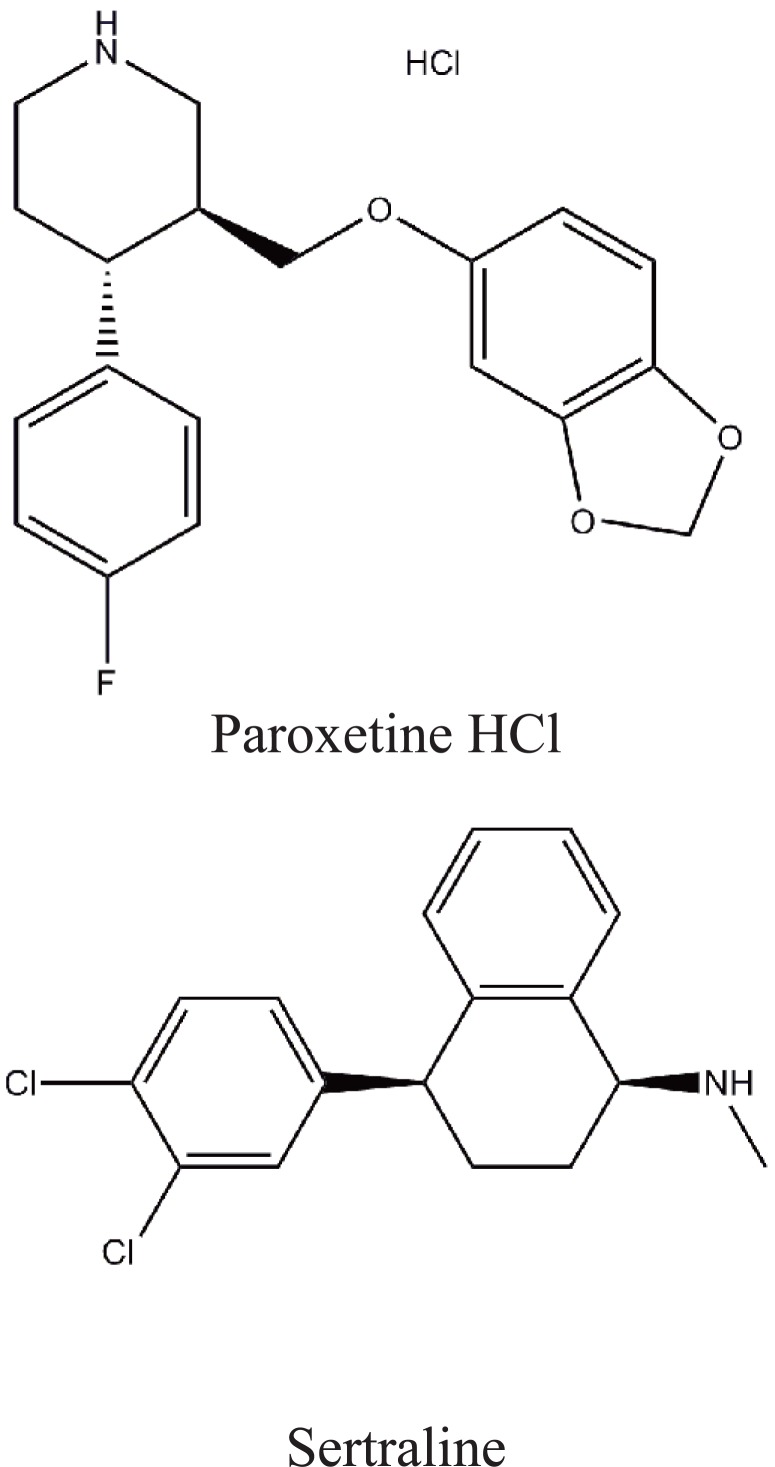
Structural formulae of the studied drugs.

Sertraline (SER) is selective serotonin reuptake inhibitor which is clinically effective for the treatment of depression, obsessive-compulsive disorders, depression relapse and social phobia (1). It is (1S,4S)-4 [3,4-dichlorophenyl]-1,2,3,4 tetrahydro-N-methyl-1-naphthylamine (Figure 1) (2). Various methods have been reported for the determination of sertraline (SER) including spectrophotometry through it’s reaction with some acidic dyes (3) and some haloquinones (4). A good deal to the work published is found in the comprehensive monographs in its analytical profile (5). It was also determined in biological fluids and dosage forms by GC MS (6-8), GC (9) and HPLC methods (10-14).

As for paroxetine (PXT), it is (3S, 4R)-3-[(1,3-Benzodioxol-5-yloxy)methyl]-4-(4-fluorophenyl) piperidine hydrochloride (15). It is a new generation antidepressant drug. It is comparable to the tricyclic antidepressants in their clinical efficacy, however, It is safer (16). It is also used in the treatment of obsessive compulsive disorder, panic fits, generalized anxiety disorders and social phobia (17). The methods reported for its determination include voltammetry (18, 19), HPLC (20-25), GC (26-28), capillary electrophoresis (29) and few spectrophotometric methods have been reported for its determination in tablets (30-33). These methods are associated with some drawbacks such as multiple extraction steps (30) and also the other reported methods (31, 32) is based on using PXT base rather than HCl salt which is more common in pharmaceutical preparations. The main goal of the study is to develop an accurate, simple and non expensive spectrophotometric method for the determination of PXT and SER in pure form and in pharmaceutical preparations.

## EXPERIMENTAL

### Apparatus

The spectrophotometric measurements were established using Shimadzu UV- Visible 1601 recording Spectrophotometer (P/N 206-67001). Recording range, 0-1.0; wavelength 390, 375 nm for SER and PXT respectively.

### Materials and reagents

All reagents and solvents were of Analytical Reagent grade.

Sertraline (SER) was kindly provided by the Egyptian Company for Chemicals and Pharmaceuticals, Cairo, Egypt. It’s purity was found to be 99.78% according to the reference method (14). Different tablet preprations were obtained from commercial sources.

Paroxetine HCl (PXT) was kindly provided by SmithKline Beecham Pharmaceuticals, Bentford, England. It’s purity was found to be 99.8% according to the reference method (33) and the tablet preprations from commercial sources.

2,4-dinitrofluorobenzene (DNFB) was purchased from (Fluka Chemie, Germany). It was freshly prepared as 0.3% v/v methanolic solution.

Borate buffer (0.2 M, pH8.0 and 9.0) was prepared by mixing appropriate volumes of 0.2 M boric acid and 0.2 M NaOH and adjusting the pH using a pH Meter. The buffer solution was kept in refrigerator and left to reach the room temperature before use.

Methanol and hydrochloric acid (32%) were purchased from (Fluka Chemie, Germany).

### Standard solution

Stock solutions were prepared by dissolving 20.0 mg of each of SER and PXT separately in 100 mL of methanol and were further diluted with the same solvent as appropriate. The standard solutions were stable for seven days when kept in the refrigerator.

## METHOD

### Recommended procedure

Transfer aliquots of SER or PXT standard solutions separately into a series of 10 mL volumetric flasks. Add 1.5 mL of borate buffer (pH8.0) in case of SER or 1 mL (pH9) in case of PXT, followed by 0.6 mL or 1 mL of 0.3% v/v DNFB for SER or PXT respectively and mix well. Heat at 60°C and 70°C for 15 and 12 min for SER and PXT respectively in a thermostatically-controlled water bath, then cool to room temperature. Add 0.2 mL of HCl and complete to the volume with methanol and mix well. Measure the absorbance of the resulting solution at 375 and 390 nm for SER and PXT respectively against a reagent blank prepared simultaneously. Plot the absorbance vs the final concentration of the drug (μg/mL) to get the calibration curves. Alternatively, derive the corresponding regression equations.

### Procedure for commercial tablets

Weigh and pulverize 10 tablets (Lustral^®^, Serlift^®^ and Sesrine^®^ for SER) or (Xandol^®^ and Paxetin^®^ for PXT). Transfer a weighed quantity of the powder equivalent to 100.0 mg of SER or PXT into a small conical flask. Extract with 3 × 30 mL of methanol on three successive times each with 30 mL. Filter the extract into a 100 mL volumetric flask. Wash the conical flask with few mls of methanol. Pass the washings into the same volumetric flask and complete to the mark with the same solvent. Transfer aliquots covering the working concentration range into 10 mL volumetric flasks. Proceed as described under “Recommended Procedure”. Determine the nominal content of the tablets either from the calibration curves or using the corresponding regression equations.

## RESULTS AND DISCUSSION

**Figure 2:**
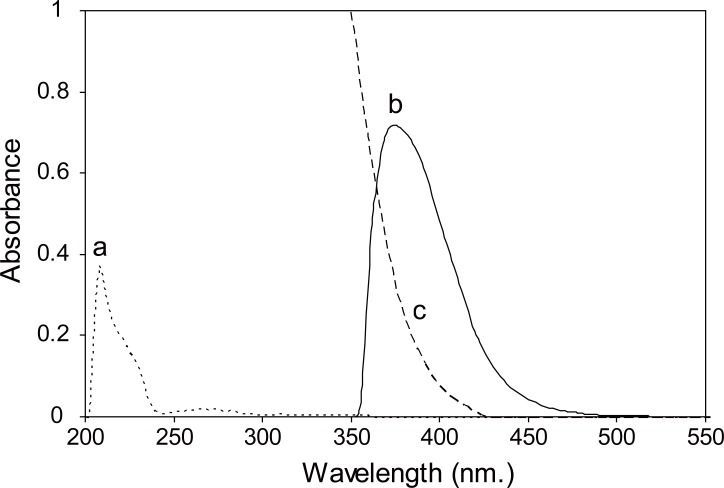
a, sertraline only (20.0 μg/mL); b, Absorption spectrum of the reaction product of sertraline (8.0 μg/mL) with 0.3% DNFB at pH8; c, Blank.

**Figure 3:**
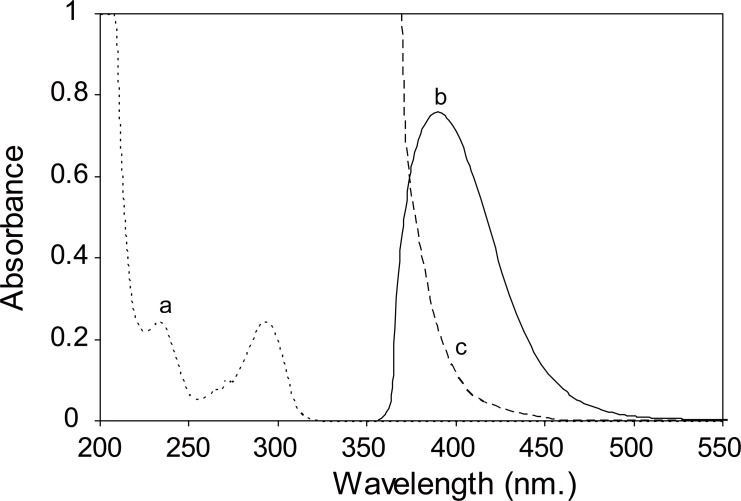
a, paroxetine HCl only (10.0 μg/mL); b, Absorption spectrum of the reaction product of paroxetine HCl (16.0 μg/mL) with 0.3% DNFB at pH9; c, Blank.

In the present study, SER and PXT were found to react with DNFB in borate buffer of pH8 and 9 producing a yellow color peaking at 375 and 390 nm for SER and PXT respectively. (Figs. 2 and 3)

The analytical applications of DNFB in the assay and characterization of amines have been established by Conner (34). The reagent had been used to quantify primary and secondary amines (35-39).

To get rid of the excess reagent interference in the absorbance measurements of the reaction product, the excess reagent is hydrolyzed to the colorless 2,4-dinitrophenol using hydrochloric acid. The exact volume was established as 0.2 mL conc. hydrochloric acid.

### Optimization of experimental parameters

The spectrophotometric properties of the colored product as well as the different experimental parameters affecting the color development and its stability were carefully studied and optimized. Such factors were changed individually while others were kept constant. The factors include pH, type of buffer, temperature, time of heating and effect of diluting solvent.

### Effect of pH

The influence of pH on the absorbance of the reaction product was evaluated. For SER, maximum absorbance intensity was obtained at pH7.8 and remained constant up to 8.2 after which the absorbance of the reaction product began to decrease gradually until pH8.5. Therefore, pH of 8.0 ± 0.2 was chosen as the optimum pH.

From the reaction of the studied drugs with DNFB a yellow colored product was formed along with the alkaline hydrolysis product of DNFB (2,4-dinitrophenolate), which exhibits an intense yellow color. The spectra of the two products are overlapped rendering impossible the measurement of the analyte derivative. However, by acidification, after the completion of the reaction, the yellow 2,4-dinitrophenolate turns to the colorless 2,4-dinitrophenol, allowing the quantitative measurement of the drugs-DNFB derivative which remains stable (40).

To remove the excess reagent interference in the absorbance measurement of the reaction product, 0.2 mL of concentrated HCl was added.

For PXT, the absorbance was maximum at pH9 ± 0.2, then at pH higher than 10 precipitation occurred. Other buffers having the same pH value such as phosphate and hexamine were tried and compared with 0.2 M borate buffer. Borate buffer was found to be superior to other buffers having the same pH value since the absorbance value was highest in case of borate buffer.

### Effect of volume of borate buffer

It was found that increasing the volume of buffer produces a gradual increase in the absorbance value of the reaction product up to 1.3 and 0.8 for SER and PXT respectively and it remaines constant up to 1.8 and 1.2 for SER and PXT respectively. Therefore, the optimum buffer volumes were 1.5 ± 0.2 and 1 ± 0.2 for SER and PXT respectively.

### Effect of volume of DNFB

The influence of the concentration of DNFB was studied using different volumes of 0.3% v/v methanolic solution of the reagent. It was found that, the reaction of DNFB with both SER and PXT started upon using 0.2 mL of the reagent. Increasing the volume of the reagent, produces a proportional increase in the absorbance of the reaction product up to 0.5 mL and remains constant up to 0.7, after which further increase produces a gradual decrease in the absorbance value. Therefore, 0.6 mL of 0.3% v/v DNFB was chosen as the optimal volume of the reagent in case of SER, but in case of PXT the increase in absorbance is gradual until 0.8 mL then the absorbance value remains constant up to 1.2 mL, after which further increase produces a gradual decrease in the absorbance value, Therefore 1 mL of 0.3% v/v DNFB was chosen as the optimum volume of the reagent.

### Effect of temperature

Different temperature settings were used with constant heating time. For SER; increasing the temperature of the water bath was found to produce a proportional increase in the absorbance of the reaction product up to 55°C and remained constant until 65°C after which further increase in the temperature produces a gradual decrease in the absorbance value, so the optimum temperature for study was 60°C ± 5°C. For PXT the optimum temperature was found to be 70°C ± 5°C.

### Effect of time

The time of heating is an essential part of the experiment. Different time intervals were tested to ascertain the time after which the solution attains its highest absorbance. It was found that the adequate heating time is 15 and 12 min for SER and PXT respectively.

### Effect of diluting solvent

Dilution with different solvents such as methanol, water, acetone, dimethylsulfoxide and dimethylformamide was studied. The highest absorbance value was achieved upon diluting with methanol for both drugs.

## ANALYTICAL PARAMETERS

### Validation of the proposed method

The validity of the methods was tested regarding linearity, specificity, accuracy, repeatability and precision according to ICH Q2B recommendations (41).

### Linearity

The absorbance-concentration plots were found to be linear over the range of 1-10 μg/mL and 2-20 μg/mL for SER and PXT respectively with minimum detection limit (LOD) of 0.11 μg/mL and 0.28 μg/mL for SER and PXT respectively. Linear regression analysis of the data gave the following equation:

A=−0.01+0.093 C  r=0.9999 for SERA=0.001+0.05 C  r=0.9999 for PXT

where A is the absorbance in 1-cm cell and C is the concentration of the drug in μg/mL.

The limits of quantification (LOQ) and the limits of detection (LOD) were calculated according to ICH Q2B (41) using the following equations:

$\mathrm{LOQ}={10S}_{a}/b\;\mathrm{LOD}={3.3S}_{a}/b$

where S_a_ is the standard deviation of the intercept of regression line, b is the slope of the calibration curve.

LOQ were found to be 0.32 and 0.85 μg/mL for SER and PXT respectively, while LOD were found to be 0.11 and 0.28 μg/mL for SER and PXT respectively.

The proposed method was evaluated by studying the accuracy as percent relative error (% Er) and it was found to be 0.26 and 0.34 for SER and PXT respectively. The precision as percent relative standard deviation (% RSD) and it was found to be 0.68 and 0.90 for SER and PXT respectively. The small values of % Er The % RSD indicates high accuracy and high precision of the proposed method.

### Accuracy

To test the validity of the proposed method it was applied to the determination of pure sample of SER and PXT over the working concentration ranges. The results obtained were in good agreement with those obtained using reference methods for both drugs respectively. Using Student’s t-test and variance ratio F-test (42), revealed no significant difference between the performance of the two methods regarding the accuracy and precision, respectively. The reference method for SER (14) involved an HPLC determination of SER in pure and dosage forms using a mixture of 35% acetonitrile : methanol (92:8, v/v) and 65% of sodium acetate buffer at pH4.5 as mobile phase with UV detection at 230 nm. The value of the tabulated t were found to be 0.29 (2.30) and 1.43 (2.23) for SER and PXT respectively. While, the values of variance ratio F test was found to be 12.1 (19.3) and 1.37 (4.53) for SER and PXT respectively. Values between parenthesis are the tabulated t and F respectively at *p*=0.05 (42). The reference method for PXT (33) is based on a spectrophotometric determination of PXT in pure and dosage forms using 1,2-Naphthoquinone-4-sulphonate as chromogenic reagent to form an orange colored product peaking at 488nm. This method obey Beer’s law limits over the concentration range of 1-8 μg/mL.

The validity of the method was evaluated by statistical analysis of the regression lines regarding the standard deviation of the residuals (S_y/x_) was found to be 3.7 × 10^-3^ and 5.23 × 10^-3^ for SER and PXT respectively, the standard deviation of the intercept (S_a_) was found to be 2.98 × 10^-3^ and 4.20 × 10^-3^ for SER and PXT respectively and standard deviation of the slope (S_b_) was found to be 4.98 × 10^-4^ and 3.51 × 10^-4^ for SER and PXT respectively. The small values of the figures points out to the low scattering of the points around the calibration graph and high precision of the method.

### Precision

**Repeatability.** The repeatability was performed by applying the proposed method for the determination of three concentrations of SER and PXT (5, 8 and 10 μg/mL) separately in pure form on three successive times. The mean % recoveries were found to be 100.06 ± 0.09 and 100.08 ± 0.68 for SER and PXT respectively.

**Intermediate precision.** It was performed through repeated analysis of SER and PXT separately in pure form, using the concentrations (5, 8 and 10 μg/mL) for a period of 3 successive days. The mean % recovery was found to be 98.82 ± 0.72 and 99.92 ± 0.22 for SER and PXT respectively.

### Robustness of the method

The robustness of the method adopted is demonstrated by the constancy of the absorbance value with the deliberated minor changes in the experimental parameters such as, change in pH 8.0 ± 0.2 for SER and 9 ± 0.2 for PXT, change in the volume of DNFB (0.3% v/v), 0.6 ± 0.1 for SER and 1.0 ± 0.2 mL for PXT and the change in reaction temperature 60°C ± 5°C for SER and 70°C ± 5°C for PXT. These minor changes that may take place during the experimental operation didn’t affect the absorbance of the reaction products.

### Pharmaceutical Applications

The proposed method was then applied to the determination of SER and PXT in their tablets. The methods were tested for linearity, selectivity, accuracy and precision according to ICH Q2B recommendations (41).

### Selectivity

The selectivity of the method was investigated by observing any interference encountered from the common tablet excepients, such as talc, lactose, starch, avisil, gelatine, and magnesium stearate. These excepients did not interfere with the proposed methods. As revealed by a placebo experiment using all tablet excipients but omitting the drug.

### Effect of co-administered drugs

The selectivity of the method was also investigated by observing any interference encountered from the co-administered drugs such as tricyclic antidepressants. It was found that there is no interference from these drugs such as: amitriptyline, clomipramine and imipramine.

### Accuracy

The results of the proposed method were statistically compared with those obtained using the reference methods (14), (33) for SER and PXT respectively. Statistical analysis (42), of the results, using Student’s t-test and Variance ratio F-test revealed no significant difference between the performance of the proposed and reference methods regarding the accuracy and precision, respectively (Table 1).

**Table 1:** Application of the proposed method to the determination of sertraline and paroxetine HCl in commercial tablets

Drug	Preparation	Spectrophotometric method		Ref. method (14, 33)	
		Amt. Taken (μg/ml)	% found	Amt. Taken (μg/ml)	% found

SER	a**Seserine^®^ tablets** (56.0 mg SER HCl/Tablet equivalent to 50.0 mg SER) B.No: 080371	2.0	98.91	0.5	100.36
		4.0	99.93	1.0	99.87
		6.0	99.05	2.0	99.56
		X ± SD	99.30 ± 0.55		99.93 ± 0.40
		t	1.602		
		F	1.870		
	b**Serlift^®^ tablets** (100.0 mg SER HCl/Tablet) B.No: 810102	2.0	101.31	0.5	99.65
		4.0	99.80	1.0	100.89
		6.0	100.53	2.0	100.25
		X ± SD	100.53 ± 0.76		100.26 ± 0.62
		t	0.502		
		F	1.480		
	c**Lustral^®^ tablets** (50.0 mg of SER/Tablet) B.No: 9201	2.0	99.341	0.5	100.23
		4.0	100.51	1.0	100.43
		6.0	100.20	2.0	98.51
		X ± SD	100.02 ± 0.61		99.72 ± 1.06
		t	0.417		
		F	3.03		
PXT	d**Paxetin^®^ tablets** (20.0 mg PXT HCl/Tablet) B.No: 1228002	5.0	101.08	2.0	100.54
		10.0	101.34	5.0	101.55
		15.0	100.57	8.0	100.34
		X ± SD	101.0 ± 0.39		100.81 ± 0.65
		t	0.427		
		F	2.70		
	e**Xandol^®^ tablets** (20.0 mg PXT HCl/tablet) B.No: 8235001	5.0	100.55	2.0	100.05
		10.0	101.84	5.0	100.98
		15.0	100.33	8.0	100.79
		X ± SD	100.91 ± 0.82		100.61 ± 0.49
		t	0.546		
		F	2.75		

The tabulated values of t and F are (2.78) and (19.00) respectively, at *p*=0.05 (41). Each result is the average of three separate determinations.

a Product of ADWIA Co S.A.E.;

b Product of GLOBAL NAPI pharmaceuticals for REXCEL EGYPT;

c Product of PFIZER EGYPT S.A.E. Cairo;

d Product of PHARAONIA pharmaceuticals;

e Product of EUROPIAN EGYPTIAN PHARM. IND.

### Mechanism of the reaction

**Figure 4:**
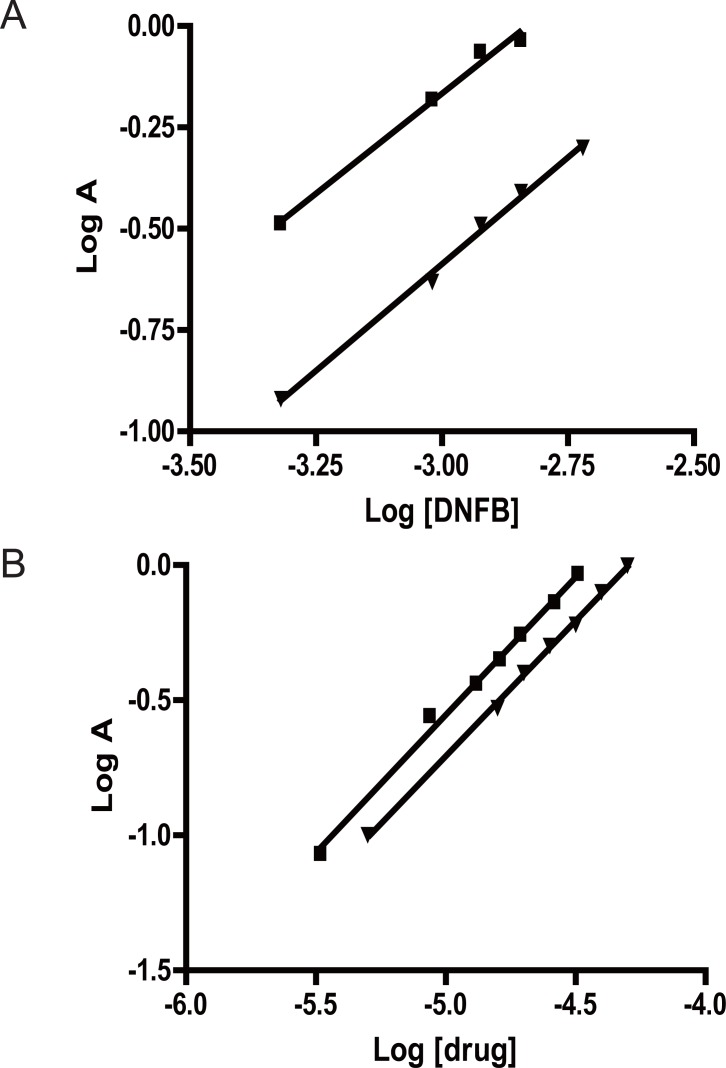
Stoichiometry of the reaction between drugs and DNFB (0.3% v/v) adopting limiting logarithmic method. A, Log [DNFB] vs log A.(■ SER and ▲PXT); B, Log [drug] vs log A.

**Figure 5:**
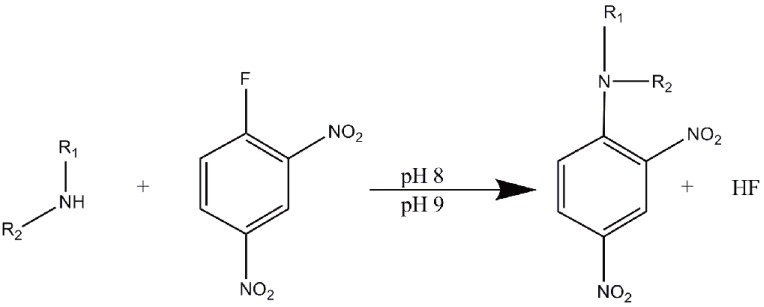
Proposed reaction pathway between DNFB and sertraline at pH=8 and at pH=9 for paroxetine.

The stoichiometry of the reaction was studied adopting the limiting logarithmic method (43). The absorbance of the reaction product was alternatively measured in the presence of excess of DNFB and either SER or PXT. A plot of log Absorbance versus log [DNFB] and log [drug] gave straight lines, the values of the slopes are 0.99 and 1.02 respectively for SER/ reagent and 1.05 and 1.00 for PXT / reagent (Fig. 4). Hence, it is concluded that, the molar reactivity of the reaction is 0.99 / 1.02 for SER and 1.05 / 1.00 for PXT, i.e. the reaction proceeds in the ratio of 1:1 in both drugs. It is confirmed that one molecule of the drug reacts with one molecule of DNFB in alkaline medium through the secondary amino group of either drugs to give the following final reaction product. A schematic proposal of the reaction pathway is represented in Figure 5.

## CONCLUSION

New simple and sensitive spectrophotometric method for the determination of SER and PXT has been successfully developed and validated. The method involved simple reaction of SER and PXT with DNFB reagent, and subsequent measuring of the absorbance of the reaction product. The proposed method could be applied without prior extraction steps for pure samples of SER and PXT respectively. Moreover, the proposed method is specific, accurate, reproducible, and highly sensitive to be applied on the analysis of tablets. Furthermore, the analysis is relied on a simple apparatus, thus the proposed method is suitable for routine analysis of SER and PXT in quality control and clinical laboratories.
